# Positive Association between Aqueous Humor Hydroxylinoleate Levels and Intraocular Pressure

**DOI:** 10.3390/molecules27072215

**Published:** 2022-03-29

**Authors:** Aya Umeno, Yasukazu Yoshida, Sachiko Kaidzu, Masaki Tanito

**Affiliations:** 1 Department of Ophthalmology, Faculty of Medicine, Shimane University, Izumo 693-8501, Japan; aumeno@med.shimane-u.ac.jp (A.U.); kecha@med.shimane-u.ac.jp (S.K.); 2 Computational Bio Big Data Open Innovation Laboratory (CBBD-OIL), National Institute of Advanced Industrial Science and Technology, Tokyo 100-0004, Japan; 3 Health Research Institute, National Institute of Advanced Industrial Science and Technology, Takamatsu 761-0301, Japan; 4 Head Office Laboratory, LG Japan Lab Inc., Kanagawa 220-0011, Japan; yasukazu.yoshida@lgjlab.com

**Keywords:** glaucoma, oxidative stress, linoleate-derived oxidation products, ocular and systemic oxidation, biomarker, pathogenesis

## Abstract

We previously proposed the total assessment of hydroxylinoleates (HODEs) by LC-MS/MS after saponification and reduction of the biologic samples as biomarkers to investigate pathogenesis, disease progression, and prognosis. In this study, HODE levels were estimated in aqueous humor (AH) samples from 63 eyes (41 Japanese subjects; 15 men; mean age, 77.3 ± 6.8 years) with primary open-angle glaucoma (POAG) or cataracts. The correlations between intraocular HODE levels and background parameters, including intraocular pressure (IOP), were analyzed to assess the possible involvement of oxidative stress in glaucoma pathology. Univariate analyses showed that linoleic acid (LA) (*p* = 0.034) and arachidonic acid (AA) (*p* = 0.0041) levels were associated negatively with age; 13-(Z,E)-HODE (*p* = 0.018) and 13-(E,E)-HODE (*p* = 0.021) were associated positively with IOP; 9-(Z,E)-HODE (*p* = 0.039), 13-(Z,E)-HODE (*p* = 0.021), totally assessed-HODE (t-HODE, *p* = 0.023), LA (*p* = 0.0080), and AA (*p* = 0.0051) were higher in eyes with glaucoma than cataract. No gender differences were seen. A mixed-effect regression model showed that higher 13-(Z,E)-HODE (*p* = 0.0040) and higher t-HODE (*p* = 0.040) were associated with glaucoma rather than cataracts; and higher levels of 13-(Z,E)-HODE/LA (*p* = 0.043), 13-(E,E)-HODE/LA (*p* = 0.042), 13-(Z,E)-HODE (*p* = 0.0054), and 13-(E,E)-HODE (*p* = 0.027) were associated with higher IOP. Linoleate-derived oxidation products were quantified successfully in AH samples from patients with glaucoma and cataracts. A free radical oxidation mechanism can be associated with IOP elevation, while enzymatic oxidation may be involved, specifically, in the pathogenesis of POAG.

## 1. Introduction

Glaucoma, which leads to visual field loss and causes irreversible blindness worldwide [1,2,3], is a progressive glaucomatous optic neuropathy. The death of retinal ganglion cells (RGCs) and RGC axon loss cause glaucomatous optic neuropathy [4]. Elevated intraocular pressure (IOP) is the primary risk factor in open-angle glaucoma, including primary open-angle glaucoma (POAG) [4]. In these OAGs, increased IOP is explained by reduced aqueous humor (AH) outflow at the trabecular meshwork (TM) due to changes in the amount and quality of the extracellular matrix in the TM [5]. Treatment using hydrogen peroxide depletion of glutathione decreases the TM outflow facility [6]. Numerous experimental studies have reported that various oxidative stresses induce RGC death [7,8,9,10]. For example, free radical scavengers prevent glaucomatous tissue injury, specifically, glutamate- and IOP-induced RGC death [11,12]. Thus, oxidative stress is likely involved in IOP elevation and RGC loss in POAG and POAG without marked IOP elevation, such as in normal-tension glaucoma [13]. We previously reported lower systemic antioxidant capacity measured by ferric-reducing activity (BAP) in serum samples from patients with POAG and glaucoma secondary to exfoliation syndrome (EXG) compared with non-glaucomatous subjects [14,15]. A comprehensive assessment reported that lower BAP levels were associated with higher IOP levels and more severe visual field damage in OAG [16,17].

**Figure 1:**
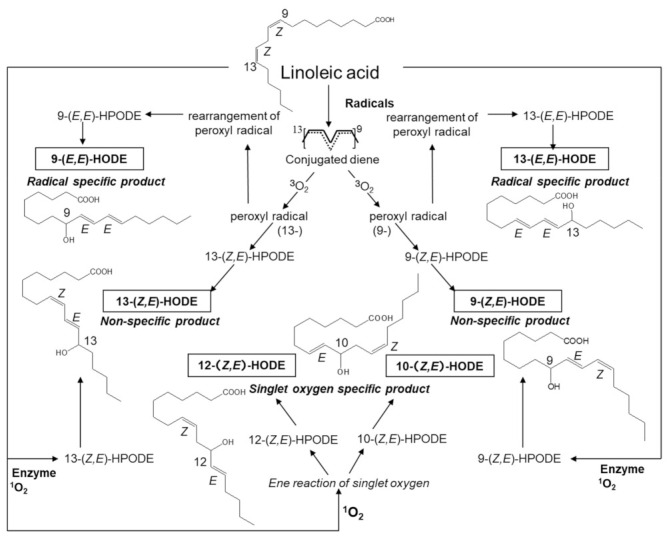
Chemical structures of HODE isomers.

We previously proposed totally assessed hydroxylinoleates (HODEs) from biologic samples as biomarkers to investigate the pathogenesis, disease progression, and prognosis [18,19,20,21]. They can provide information on the effectiveness of functional factors for disease prevention. The advantage of measuring oxidation products of linoleates is that they are the most abundant polyunsaturated fatty acids in vivo and their oxidation proceeds by a straightforward mechanism that yields much simpler products than arachidonates and more highly unsaturated fatty acids, such as docosahexaenoates [22]. HODEs are formed by a radical-mediated oxidation mechanism that consists of four isomers, i.e., 13-(Z,E)-HODE, 13-(E,E)-HODE, 9-(Z,E)-HODE, and 9-(E,E)-HODE (Figure 1). Little 11-HODE is formed under normal conditions as the pentadienyl radical that is formed by the abstraction of hydrogen at the 11-carbon, which rearranges rapidly to form stable conjugated diene radicals. Enzymatic oxidation also forms 9- and 13-(Z,E)-HODEs via lipoxygenase as enantio-, regio-, and stereo-specific products [23]. Thus, 9- and 13-(E,E)-HODEs are specific products of radical-mediated oxidation. However, singlet oxygen and ozone oxidize linoleic acids (LAs) form 13-(Z,E)-HODE and 9-(Z,E)-HODE by non-radical oxidation. The absolute concentrations of lipid hydroperoxides in vivo are considered minimal since they are substrates of many enzymes, such as glutathione peroxidases and phospholipases. In such cases, the stable oxidation products are HODEs. Linoleates are more stable than arachidonates and docosahexaenoates regarding free-radical-mediated oxidation. The implications of using HODEs as biomarkers for detecting early-stage diseases were reviewed previously [23]. We reported that the serum levels of 9- and 13-(Z,E)-HODEs increased in POAG, compared with controls based on a comprehensive assessment [24]. However, in EXG, 9- and 13-(E,E)-HODE levels were significantly higher than the controls, suggesting the different roles of (Z,E)- and (E,E)-HODEs between POAG and EXG [25]. Both studies indicated that systemic free-radical-mediated oxidation is involved in the deterioration of POAG and EXG, and that LA oxidation products may serve as biomarkers for this pathology.

We previously showed in POAG patient samples that the BAP serum levels were correlated negatively with superoxide dismutase activity in the AH [26], suggesting that systemic oxidative stress reflects the intraocular redox status. In the current study, to assess more directly the possible involvement of oxidative stress in levels of IOP, the HODE levels were estimated in AH samples from eyes with POAG and cataracts, and the correlations between intraocular HODE levels and background parameters, including IOP, were statistically analyzed.

## 2. Results

The subject-based and eye-based demographic data are summarized in Table 1. In this dataset, the mean IOP was 14.3 mmHg (range, 4–25 mmHg). The measured HODEs, LA, and arachidonic acid (AA) in the AH are summarized in Table 2. In this study, only subjects who did not use topical medications were included; therefore, no subjects used anti-glaucoma medications.

The possible associations between HODEs and demographic parameters were assessed by univariate analyses. For continuous variables (Table 3), LA (*p* = 0.034) and AA (*p* = 0.0041) levels were associated negatively with age; 13-(Z,E)-HODE (*p* = 0.018) and 13-(E,E)-HODE (*p* = 0.021) were associated positively with IOP. For categorical variables (Table 4), 9-(Z,E)-HODE (*p* = 0.039), 13-(Z,E)-HODE (*p* = 0.021), t-HODE (*p* = 0.023), LA (*p* = 0.0080), and AA (*p* = 0.0051) were higher in eyes with glaucoma than cataracts; meanwhile, none differed between men and women.

We further assessed the association between HODEs and demographic parameters using a mixed-effect regression model (Table 5). The results showed that higher 13-(Z,E)-HODE (*p* = 0.0040) and higher t-HODE (*p* = 0.040) were associated with glaucoma rather than cataracts; higher 13-(Z,E)-HODE/LA (*p* = 0.043), higher 13-(E,E)-HODE/LA (*p* = 0.042), higher 13-(Z,E)-HODE (*p* = 0.0054), and higher 13-(E,E)-HODE (*p* = 0.027) were associated with higher IOP.

## 3. Discussion

Our method for measuring lipid-derived oxidation products by LC-MS/MS after saponification and reduction of the biologic samples allows direct quantitative assessment of large amounts of oxidation products from linoleates and arachidonates. This method also provides the total amounts of linoleates and arachidonates as free fatty acids at a time. Among them, linoleate-derived oxidative stress markers are advantageous because they facilitate an understanding of the oxidative mechanism in diseases, based on their isomeric distributions. We have previously revealed that oxidative stress is involved in the pathogenesis of several diseases, such as diabetes, Alzheimer’s disease, and Parkinson’s disease, by measuring HODEs [22,23]. Notably, it was found that singlet oxygen is strongly correlated with the onset of diabetes on the basis of specific biomarkers, 10- and 12-(Z,E)-HODEs [23]. We believe that biomarkers of HODEs are a useful tool for clarification of pathological mechanisms.

We successfully measured the HODE levels in AH and found that the levels were three orders of magnitude lower than serum levels [24,25]. This was reasonable because the levels of the parent molecules, LA and AA, were also quite low (Table 2), more than three orders of magnitude lower than those in serum [24,25]. It is interesting that the levels of HODEs/LA in AH were similar to the serum levels (Table 2), suggesting that the oxidation in the eye proceeds at the same pace as systemic oxidative stress. Strikingly, 13-(Z,E) and (E,E)-HODEs were correlated significantly with the IOP (Table 3), which strongly indicates that oxidative stress is, indeed, involved in the pathogenesis of the disease. To our knowledge, this study is the first to report the direct contents of oxidized LA products from the AH samples. It is also interesting that the levels of LA and AA correlated with age (Table 3), possibly due to the decreased antioxidative defense system, but the reason should be clarified further in a future study. For categorical variables, the levels of 9- and 13-(Z,E)-HODE and tHODE were higher in glaucoma than those in cataracts, suggesting that oxidative stress in glaucoma proceeds further than cataracts. On the other hand, the levels of LA and AA in glaucoma were also higher than those in cataracts. One possible reason is the adaptive response toward oxidative stress, which should be clarified by a future study. Unfortunately, neither singlet oxygen-specific oxidation products, 10- and 12-(Z,E)-HODEs, nor arachidonate-derived oxidation products, such as hydroxyarachidonates and isoprostanes, were detected quantitatively.

The systemic status of redox (reduction/oxidation) in glaucoma has been a subject of interest after the identification of the circulating autoantibodies against antioxidative stress enzymes and chaperone molecules glutathione S-transferase [27], and heat shock proteins [28,29] in the serum of patients with glaucoma. A number of studies have reported on this topic [30,31,32]. Our previous work showed that enzymatic and singlet oxygen-mediated oxidation may be major pathways in POAG, based on the increase in 9- and 13-(Z,E)-HODEs and 10- and 12-(Z,E)-HODEs in patient serum samples [24]. In the current study, univariate analyses showed that the levels of 9- and 13-(Z,E)-HODEs and t-HODEs in the AH from POAG eyes were significantly higher than those in cataractous eyes (Table 4). Multivariate analysis showed that the levels of 13-(Z,E)- and t-HODEs were higher in POAG than cataracts again, suggesting that enzymatic oxidation is involved intraocularly in this disease, which is consistent with our previous systemic evaluation [24]. Univariate analysis showed that higher 13-(Z,E) and 13-(E,E)-HODEs levels were associated with higher IOP (Table 3). Multivariate analysis indicated that 13-(Z,E) and (E,E)-HODEs and 13-(Z,E) and (E,E)-HODEs/LA were correlated significantly with IOP again (Table 5). Previously, in EXG, 9- and 13-(E,E)-HODEs increased according to the pathogenesis of the exfoliation syndrome, suggesting the involvement of free-radical-mediated oxidation in this syndrome [25]. Collectively, the results suggest the possible involvement of (Z,E)-HODEs, specifically in the POAG pathogenesis, while the (E,E)-HODEs are involved in IOP elevations, which is commonly seen in various glaucoma types. Oxidative stress-induced DNA damage was detected in the TM tissues from POAG and EXG [33]; thus, the free radical damage to the TM cells can be an underlying mechanism of the positive correlation between (E,E)-HODEs and IOP in our study. Furthermore, the increase of (E,E)-HODE and IOP were correlated negatively with SOD in AH, measured in our previous work, suggesting the elevation and deterioration of oxidative stress and antioxidant defense system, respectively.

This study included a relatively small number of glaucoma patients. However, these patients did not use topical medication or anti-glaucoma medications. It is very important to clarify the pathological mechanism. In fact, we have collected more than 265 AH samples from cataract and glaucoma patients, but more than 80% patients underwent medications. The use of a beta-adrenergic receptor blocker or prostaglandins, major ocular hypotensive agents, can affect the intraocular lipid profile [34,35]. Therefore, we included the AH samples from the eyes of patients who did not use any topical medications. With this inclusion criterion, the current results were free from the effects of topical medications; instead, the number of eyes with POAG was limited, and advanced glaucoma might be excluded from the study. The inclusion of both eyes of a patient may have introduced bias, although we minimized this by using a mixed-effects regression model. We believe that the study design is scientifically reasonable to explore possible correlations between IOP and HODEs levels in the AH.

## 4. Materials, Subjects, and Methods

### 4.1. Materials

Lipid-peroxidation products, such as 9-(Z,E)-HODE, 13-(Z,E)-HODE, 9-(E,E)-HODE, 13-(E,E)-HODE, and the internal standard, 13S-hydroxy-10*E*,12*Z*-octadecadienoic-9,10,12,13-*d*_4_ acid (13-HODE-*d*_4_), were obtained from Larodan Fine Chemicals (Malmo, Sweden); LA and AA were obtained from Sigma–Aldrich (St. Louis, MO, USA). Other materials were of the highest commercially available grade.

### 4.2. Subjects

A total of 63 eyes of 41 Japanese subjects (77.3 ± 6.8 years; 15, 37% men and 26, 63% women; 49 eyes of 32 subjects with cataracts and 14 eyes of 9 subjects with POAG) were recruited consecutively at Shimane University Hospital, Shimane, Japan. All measurements of oxidative stress markers were conducted at the National Institute of Advanced Industrial Science and Technology. The current study adhered to the tenets of the Declaration of Helsinki. The institutional review boards (IRBs) of Shimane University Hospital (IRB Nos. 20071227-1 and 20131216-1) reviewed and approved the research. All subjects provided written, informed consent. All subjects underwent ophthalmologic examinations, including measurements of IOP by Goldmann applanation tonometry and slit-lamp, gonioscopic, and funduscopic examinations under pupillary dilatation, as described previously [14]. POAG was diagnosed based on the presence of an open iridocorneal angle bilaterally, the characteristic appearance of glaucomatous optic neuropathy such as enlargement of the optic disc cup or focal thinning of the neuroretinal rim, corresponding visual field defects identified using the Humphrey Visual Field Analyzer (Carl Zeiss Meditec, Dublin, Ireland) in at least one eye, and the absence of secondary glaucoma bilaterally. The patients with cataracts had no glaucomatous optic neuropathy or history of an IOP of 21 mmHg or higher in both eyes. Except for a cataract and/or glaucoma, no subjects had ocular pathologies, such as clinically detectable ocular inflammation, infection, neuropathies, retinopathies, or maculopathies. To avoid any effects of topically applied medications, patients using anti-glaucoma or other topical medications were not included in the study.

### 4.3. AH Samples

At the beginning of glaucoma or cataract surgery, AH samples were obtained by aspiration through a limbal paracentesis using a 0.5-mL syringe with a 30-gauge needle (BD Japan, Tokyo, Japan). During the sampling, care was taken to prevent contamination of blood and intraocular tissues [35]. AH samples were frozen with liquid nitrogen immediately after the sampling, and then stored at −80 °C until the analyses. All patients were prescribed topical 0.5% levofloxacin four times daily for 3 days preoperatively. Patients who underwent cataract surgery, or combined cataract and glaucoma surgery, received the following drops: 1% tropicamide, 5% phenylephrine, and 0.4% oxybuprocaine every 30 min for 2 h preoperatively. Patients who underwent glaucoma surgery alone received 2% pilocarpine and 0.4% oxybuprocaine drops every 30 min for 2 h preoperatively.

### 4.4. Analysis of Oxidative Stress Markers

The levels of HODEs were measured as described previously [20] with slight modifications. The parent molecules, i.e., LAs and AAs, were detected using the same protocol: 20–180 μL of AH were mixed with saline to make 500 μL of each sample and 500 μL of methanol containing the internal standards 13-HODE-*d*_4_, and 100 μM of butylated hydroxytoluene were added to the samples. This was followed by hydroperoxide reduction using an excess of triphenylphosphine and saponification with 1 M KOH in methanol. The mixture was acidified with 10% acetic acid in water and extracted with chloroform and ethyl acetate. Each sample was then evaporated to dryness under nitrogen and the derivatized sample was reconstituted with methanol and subjected to LC-MS/MS analysis of HODEs. An aliquot of the sample was analyzed by LC-MS/MS on a TSQ Quantum Access Max system (Thermo Fisher Scientific, Waltham, MA, USA). The Dionex Ultimate 3000 system (Thermo Fisher Scientific) used for high-performance liquid chromatography consisted of an HPG-3400 RS pump, WPS-3000 TPL RS Well Plate autosampler, and TCC-3000 RS column compartment equipped with a Hypersil Gold ODS column (3.0 μm, 100 mm × 2.1 mm, Thermo Fisher Scientific) set at 40 °C. Elution was performed using a gradient of solvent A (2 mM ammonium acetate in water) and solvent B (methanol:acetonitrile = 5:95) at a flow rate of 0.2 mL/min. The initial gradient composition was 80% A and 20% B. The composition was changed to 79% A and 21% B for 10 min and then to 0% A and 100% B for 45 min. Electrospray ionization was performed at a needle voltage of 3.0 kV. Nitrogen was used as the sheath gas (50 psi) and auxiliary gas (10 units). The capillary was heated to 300 °C and the mass spectrometer was optimized for maximal sensitivity. A specific precursor-to-product ion transition was achieved by monitoring selected reactions after collision-induced dissociation in negative mode. Argon was used as the collision gas, and the collision cell pressure was 1.5 mTorr. The precursor, product ions, and collision energy were determined after MS/MS optimization as follows: *m*/*z* = 295.0 and 195 at 18 eV for both 13-(Z,E)-HODE and 13-(E,E)-HODE; *m*/*z* = 295.0 and 171 at 18 eV for both 9-(Z,E)-HODE and 9-(E,E)-HODE; *m*/*z* = 198 at 18 eV for 13-HODE-d_4_; *m*/*z* = 279 at 5 eV for LA; and *m*/*z* = 303 and 259 at 12 eV for AA.

### 4.5. Statistical Analysis

Possible correlations between background parameters and each of the oxidative stress markers (HODEs) were assessed by linear regression analysis, with Pearson’s correlation coefficient for continuous variables (i.e., age and IOP), and by the unpaired t-test for categorical variables (i.e., sex and disease type). To adjust for possible biases derived from the inclusion of both eyes of a patient, the correlation between background parameters and each of the oxidative stress markers (HODEs) were assessed using a mixed-effects regression model in which each patient’s identification number was regarded as a random effect and the background parameters (i.e., age, sex, disease type, and IOP) as a fixed effect. We performed all statistical analyses using JMP Pro statistical software version 15.2 (SAS Institute, Inc., Cary, NC, USA). All reported *p* values are two-sided. The data are expressed as the means ± standard deviation for continuous variables and in numbers and percentage for categorical variables.

## 5. Conclusions

Linoleate-derived oxidation products were quantified successfully in AH samples from patients with glaucoma and cataracts. Provided the levels of them were three orders of magnitude lower than serum levels, the methods described in this manuscript are with sensitivity high enough for analyzing various biological samples. By univariate and/or multivariate analysis, 9- and 13-(Z,E) HODEs and tHODE were higher in POAG than cataracts, while 13-(Z,E) and 13-(E,E) HODEs were positively associated with IOP, suggesting that a free radical oxidation mechanism can be associated with IOP elevation (e.g., TM damage), while enzymatic oxidation may be involved specifically in the pathogenesis of POAG (e.g., RGC damage). The current AH sample study and our previous serum sample studies have shown the involvements of oxidation products of linoleates in glaucoma pathogenesis. Thus, the measuring of HODEs in biological samples seems promising as the disease markers of glaucoma.

## Figures and Tables

**Table 1:** Demographic subject data.

Parameter	Mean ± SD or *N* (%)	95% CI or *N* (%)
Subjects (*N* = 41)		
Age, years	77.3 ± 6.8	75.2, 79.5
Sex	Men, 15 (36.6)	Women, 26 (63.4)
Disease type	Cataract, 32 (78.0)	Glaucoma, 9 (21.0)
Eyes (*N* = 63)		
Age, years	77.4 ± 6.8	75.6, 79.1
Sex	Men, 23 (36.5)	Women, 40 (63.5)
Disease type	Cataract, 49 (77.8)	POAG, 14 (22.2)
IOP, mmHg	14.3 ± 3.1	13.5, 15.1

SD, standard deviation; 95% CI, 95% confidence interval range; IOP, intraocular pressure; *N*, number.

**Table 2:** HODE levels in AH.

Parameter	Mean ± SD	95% CI
9-(Z,E)-HODE/LA, µmol/mol	243.0 ± 187.7	195.8, 290.3
9-(E,E)-HODE/LA, µmol/mol	55.0 ± 55.7	41.0, 69.1
13-(Z,E)-HODE/LA, µmol /mol	194.6 ± 198.3	144.7, 244.6
13-(E,E)-HODE/LA, µmol/mol	30.2 ± 25.0	23.9, 36.5
t-HODE/LA, µmol/mol	522.9 ± 413.6	418.7, 627.0
9-(Z,E)-HODE, nM	0.89 ± 0.60	0.74, 1.04
9-(E,E)-HODE, nM	0.20 ± 0.16	0.16, 0.24
13-(Z,E)-HODE, nM	0.71 ± 0.68	0.54, 0.88
13-(E,E)-HODE, nM	0.11 ± 0.08	0.09, 0.13
t-HODE, nM	1.91 ± 1.26	1.57, 2.25
LA, mM	0.0052 ± 0.0039	0.0042, 0.0062
AA, mM	0.00044 ± 0.00036	0.00035, 0.00053

SD, standard deviation; 95% CI, 95% confidence interval range; HODE, hydroxylinoleate; t-HODE, total HODE; LA, linoleic acid; AA, arachidonic acid.

**Table 3:** Possible association between HODE levels and age or IOP.

Parameter	Age, per Year			IOP, per mmHg		
	Estimate	95% CI	*p* Value	Estimate	95% CI	*p* Value
9-(Z,E)-HODE/LA, µmol/mol	2.2	−4.9, 9.2	0.54	6.3	−9.1, 21.7	0.42
9-(E,E)-HODE/LA, µmol/mol	0.6	−1.5, 2.7	0.58	−0.8	−5.4, 3.8	0.72
13-(Z,E)-HODE/LA, µmol /mol	5.0	−2.4, 12.3	0.18	14.6	−1.3, 30.6	0.07
13-(E,E)-HODE/LA, µmol/mol	0.7	−0.2, 1.7	0.12	1.9	−0.1, 3.9	0.06
t-HODE/LA, µmol/mol	8.4	−6.9, 23.8	0.28	22.0	−11.6, 55.7	0.20
9-(Z,E)-HODE, nM	0.00	−0.02, 0.02	>0.99	0.03	−0.02, 0.08	0.25
9-(E,E)-HODE, nM	0.00	0.00, 0.01	0.57	−0.01	−0.02, 0.01	0.42
13-(Z,E)-HODE, nM	0.01	−0.02, 0.03	0.61	0.07	0.01, 0.12	0.018 *
13-(E,E)-HODE, nM	0.00	0.00, 0.00	0.48	0.01	0.00, 0.01	0.021 *
t-HODE, nM	0.01	−0.04, 0.06	0.71	0.10	−0.01, 0.20	0.09
LA, mM	−0.0002	−0.0003, −0.0000	0.034 *	−0.0001	−0.0004, 0.0002	0.48
AA, mM	−0.00002	−0.00003, −0.00001	0.0041 **	0.00000	−0.00003, 0.00003	0.95

*p* values are calculated using linear regression analysis. * *p* < 0.05, ** *p* < 0.01. IOP, intraocular pressure; SD, standard deviation; 95% CI, 95% confidence interval range; HODE, hydroxylinoleate; t-HODE, total HODE; LA, linoleic acid; AA, arachidonic acid.

**Table 4:** Possible association between HODE levels and sex or disease type.

Parameter	Sex			Disease		
	Men	Women	*p* Value	Cataract	Glaucoma	*p* Value
9-(Z,E)-HODE/LA, µmol/mol	216.8 ± 178.0	258.1 ± 193.6	0.40	247.5 ± 171.7	227.5 ± 242.5	0.73
9-(E,E)-HODE/LA, µmol/mol	46.6 ± 37.9	59.9 ± 63.7	0.37	57.6 ± 59.3	46.2 ± 41.3	0.51
13-(Z,E)-HODE/LA, µmol /mol	189.6 ± 218.1	197.5 ± 188.8	0.88	183.9 ± 149.3	232.1 ± 321.4	0.43
13-(E,E)-HODE/LA, µmol/mol	26.9 ± 21.5	32.0 ± 27.0	0.44	31.0 ± 23.2	27.3 ± 31.5	0.63
t-HODE/LA, µmol/mol	480.0 ± 413.9	547.5 ± 416.7	0.54	519.9 ± 343.2	533.1 ± 617.2	0.92
9-(Z,E)-HODE, nM	1.01 ± 0.74	0.82 ± 0.49	0.25	0.81 ± 0.53	1.18 ± 0.74	0.039 *
9-(E,E)-HODE, nM	0.23 ± 0.18	0.18 ± 0.15	0.23	0.19 ± 0.17	0.24 ± 0.16	0.32
13-(Z,E)-HODE, nM	0.83 ± 0.85	0.64 ± 0.56	0.29	0.61 ± 0.53	1.08 ± 1.00	0.021 *
13-(E,E)-HODE, nM	0.12 ± 0.08	0.11 ± 0.08	0.56	0.10 ± 0.07	0.13 ± 0.10	0.21
t-HODE, nM	2.19 ± 1.67	1.75 ± 1.13	0.22	1.71 ± 1.13	2.63 ± 1.82	0.023 *
LA, mM	0.0060 ± 0.0042	0.0047 ± 0.0037	0.23	0.0045 ± 0.0034	0.0076 ± 0.0047	0.0080 **
AA, mM	0.00051 ± 0.00042	0.00040 ± 0.00032	0.24	0.00038 ± 0.00029	0.00067 ± 0.00047	0.0051 **

*p* values are calculated using an un-paired *t*-test. * *p* < 0.05, ** *p* < 0.01. Data are expressed as the mean ± standard deviation. HODE, hydroxylinoleate; t-HODE, total HODE; LA, linoleic acid; AA, arachidonic acid.

**Table 5:** Possible association between HODE levels and background parameters by multivariate analysis.

Parameter	Age, per Year			Sex, Men/Women			Disease, Glaucoma/Cataract			IOP, per mmHg		
	Estimate	95% CI	*p* Value	Estimate	95% CI	*p* Value	Estimate	95% CI	*p* Value	Estimate	95% CI	*p* Value
9-(Z,E)-HODE/LA, µmol/mol	2.8	−6.4, 12.0	0.54	−20.5	−86.0, 44.9	0.53	8.9	−62.7, 8.6	0.80	6.4	−12.1, 24.9	0.49
9-(E,E)-HODE/LA, µmol/mol	0.3	−2.3, 2.9	0.80	−4.9	−23.4, 13.7	0.60	−1.5	−22.2, 19.2	0.89	−0.5	−5.8, 4.8	0.84
13-(Z,E)-HODE/LA, µmol /mol	8.3	−0.1, 16.8	0.05	−16.6	−77.5, 44.3	0.58	49.0	−20.1, 118.1	0.16	18.3	0.6, 36.0	0.043 *
13-(E,E)-HODE/LA, µmol/mol	0.9	−0.2, 2.0	0.11	−3.1	−11.2, 4.9	0.44	1.5	−7.4, 10.4	0.74	2.4	0.1, 4.7	0.042 *
t-HODE/LA, µmol/mol	13.3	−6.3, 32.9	0.18	−39.6	−179.6, 100.4	0.57	59.3	−95.2, 213.7	0.44	24.4	−15.4, 64.2	0.22
9-(Z,E)-HODE, nM	0.02	−0.01, 0.05	0.28	0.07	−0.02, 0.28	0.52	0.21	−0.02, 0.44	0.08	0.01	−0.04, 0.07	0.62
9-(E,E)-HODE, nM	0.00	−0.00, 0.01	0.32	0.03	−0.02, 0.09	0.21	0.03	−0.03, 0.09	0.36	−0.01	−0.02, 0.01	0.30
13-(Z,E)-HODE, nM	0.01	−0.01, 0.03	0.19	−0.07	−0.21, 0.08	0.35	0.28	010, 0.45	0.0040 **	0.06	0.02, 0.11	0.0054 **
13-(E,E)-HODE, nM	0.00	−0.00, 0.01	0.13	−0.00	−0.03, 0.02	0.73	0.02	−0.01, 0.05	0.15	0.01	0.00, 0.02	0.027 **
t-HODE, nM	0.05	−0.01, 0.12	0.10	0.14	−0.33, 0.60	0.55	0.54	0.03, 1.05	0.040 *	0.08	−0.05, 0.21	0.22
LA, mM	−0.0001	−0.0003, 0.0000	0.13	0.0005	−0.0008, 0.0018	0.41	0.0000	−0.0010, 0.0011	0.96	−0.0002	−0.0004, 0.0001	0.21
AA, mM	−0.00002	−0.00003, 0.00000	0.05	0.00001	−0.00010, 0.00012	0.84	0.00004	−0.00004, 0.00013	0.32	−0.00000	−0.00002, 0.00002	0.89

*p* values are calculated using a mixed-effect regression analysis. * *p* < 0.05, ** *p* < 0.01. IOP, intraocular pressure; 95% CI, 95% confidence interval range; HODE, hydroxylinoleate; t-HODE, total HODE; LA, linoleic acid; AA, arachidonic acid.

## Data Availability

The data are fully available upon reasonable request to the corresponding author.
